# Quantitative Determination of Flavonoids and Chlorogenic Acid in the Leaves of *Arbutus unedo* L. Using Thin Layer Chromatography

**DOI:** 10.1155/2013/385473

**Published:** 2013-07-31

**Authors:** Željan Maleš, Darija Šarić, Mirza Bojić

**Affiliations:** ^1^University of Zagreb, Faculty of Pharmacy and Biochemistry, A. Kovačića 1, 10000 Zagreb, Croatia; ^2^Agency for Medicinal Products and Medical Devices of Croatia, Ksaverska cesta 4, 10000 Zagreb, Croatia

## Abstract

The plant species *Arbutus unedo* shows numerous beneficial pharmacological effects (antiseptic, antidiabetic, antidiarrheal, astringent, depurative, antioxidant, antihypertensive, antithrombotic, and anti-inflammatory). For the medicinal use, standardization of extracts is a necessity, as different compounds are responsible for different biological activities. In this paper, we analyze monthly changes in the content of quercitrin, isoquercitrin, hyperoside, and chlorogenic acid. Methanolic extracts of the leaves are analyzed by HPTLC for the identification and quantification of individual polyphenol, and DPPH test is used to determine antioxidant activity. Based on the results obtained, the leaves should be collected in January to obtain the highest concentrations of hyperoside and quercitrin (0.35 mg/g and 1.94 mg/g, resp.), in June, July, and October for chlorogenic acid (1.45–1.46 mg/g), and for the fraction of quercitrin and isoquercitrin in November (1.98 mg/g and 0.33 mg/g, resp.). Optimal months for the collection of leaves with the maximum recovery of individual polyphenol suggested in this work could direct the pharmacological usage of the polyvalent herbal drugs.

## 1. Introduction

*Arbutus unedo* L. (Ericaceae, English strawberry tree) is an evergreen shrub or a small tree reaching up to 12 m in height. It is found mainly in European Mediterranean region growing in maquis, evergreen scrub, woodland margins, and on rocky slopes. The leaves of *A. unedo* are alternate, simple, oblanceolate, dark green, leathery, short-stalked, and toothed. The flowers are bell shaped, with recurved lobes, 8-9 mm long, white, often tinged with pink or green, and honey scented. The fruits are globose berries about 15–20 mm in diameter, ripening through yellow to scarlet and deep crimson. Since the fruits take about 12 months to ripen, a tree carries mature fruits and flowers at the same time, and the appearance of both during winter months also makes this plant very popular for specimen plantings [1, 2].

The leaves of *A. unedo* are used as a urinary antiseptic, antidiabetic, antidiarrheal, astringent, depurative, antioxidant, antihypertensive, antithrombotic, anti-inflammatory agent [3–8]. Chemical investigations of leaves and fruits show the presence of essential oil, flavonoids, proanthocyanidins, iridoid glucosides, sugars, nonvolatile and phenolic acids, vitamins C and E and carotenoids [9–13].

As a pharmacological activity can rarely be attributed to a group of compounds as it is the case with polyphenols and antioxidant activity, the identification and quantification of individual compounds responsible for a biological activity are of interest. The objective of this paper was the identification and quantification of chlorogenic acid and flavonoids: quercitrin, isoquercitrin, and hyperoside using a simple thin layer chromatography technique.

## 2. Experimental

### 2.1. Plant Materials, Reagents, Chemicals, and Solutions

Each month in the year of 2003 the leaves of ten *A. unedo* plants were collected on five different locations on the island of Dugi otok, Božava municipality (44° 8′ 30′′ N, 14° 54′ 30′′ E). Voucher specimens (no. 99450-99461) were deposited at the Department of Pharmaceutical Botany, Faculty of Pharmacy and Biochemistry, University of Zagreb. Solvents of the analytical grade were obtained from Kemika (Croatia) and standards (quercitrin, isoquercitrin, hyperoside, and chlorogenic acid) were purchased from C. Roth (Germany). 2,2-Diphenyl-1-picrylhydrazyl was supplied by Sigma-Aldrich (USA) and HPTLC silica gel 60 F254 by Merck (Germany).

### 2.2. Sample and Standard Preparation

Extracts of *A. unedo* were prepared by the reflux extraction of leaves powder in methanol for 5 minutes; final concentration being 0.1 g/mL. The standards of polyphenols were prepared as 1 mg/mL solutions in methanol.

### 2.3. Thin Layer Chromatography

Thin layer chromatography was performed on 10 × 20 cm HPTLC silica gel 60 F254 plates (Merck, Germany). Ethyl acetate-formic acid-acetic acid-water in volume ratio 100 : 11 : 11 : 26 was used as mobile phase [14]. After development plates were air dried and recorded at 254 and 366 nm, identification and quantification were performed by TLC densitometry using CAMAG TLC Scanner 3 and WinCATS software version 1.3.4 (Switzerland). Quantification was performed using calibration curves (peak area of chromatogram versus mass of standard applied in the form of band) for individual standard in triplicate.

### 2.4. DPPH Test

Antioxidant activity was assessed using stable free radical 2,2-diphenyl-1-picrylhydrazyl (DPPH). DPPH solution was prepared by dissolving DPPH in ethanol to obtain the final concentration of 0.3 mM. Decolorization of DPPH in the presence of extract (100 : 1 volume ration) was measured on Varian Cary 50 Bio spectrophotometer (USA). Antioxidant activity (AA) was expressed as a percentage of quenching of the stable free radical at *λ* = 518 nm as follows:

(1) $$\begin{array}{c} \text{AA}=\frac{\left(A_{0}-A\right)}{A_{0}}\;\times 100, \end{array}$$

where *A*_0_ represents the absorbance of blank (methanol) and *A* absorbance of the extract measured 1 minute after mixing.

## 3. Results and Discussion

Qualitative analysis of polyphenols in leaves and fruits of *A. unedo* [14] showed presence of nine bands in the methanol extracts. Seven polyphenol standards were used and four flavonoids and chlorogenic acid were identified, out of which we were able to determine the content (quantify) of chlorogenic acid, quercitrin, isoquercitrin, and hyperoside.

Identification of each polyphenol was based on the color of the band observed under *λ* = 366 nm, *R*_*F*_ value, and matching UV-Vis spectra *in situ* with the standard used (Table 1).

Volumes of extracts applied to the plate were adjusted to correspond to ranges of linearity for individual polyphenol that were 0.2–1.6 *μ*g per band for chlorogenic acid, 1.0–5.0 *μ*g per band for quercitrin, 2.5–12.5 *μ*g per band for isoquercitrin, and 0.2–0.6 *μ*g per band for hyperoside. The results of HPTLC quantification of an individual polyphenol are presented in Table 2.

Compared to the results of the total flavonoids of 1.30–2.00 g per 100 g of dried leaves from our previous study [14], quercitrin, isoquercitrin, and hyperoside (2.38 mg/g—sum of individual flavonoids for June, Table 2) can be accounted for up to 12% of dry powder. However, it should be noticed that methods for determination of total flavonoids usually use hydrolyzed extracts, meaning that aglycones are analyzed, whereas all the analyzed polyphenols in this work present flavonoid glycosides. 

**Figure 1:**
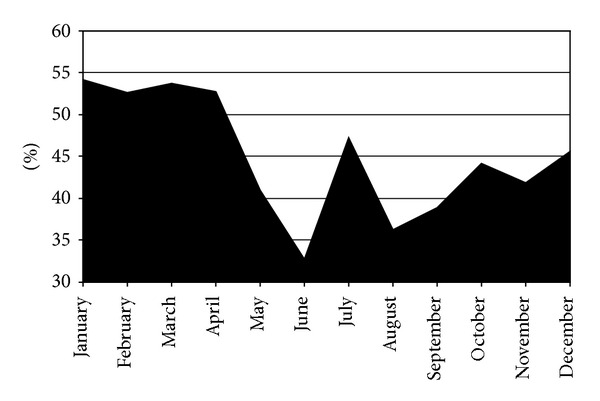
Variation of antioxidant activity expressed in percentages during the year.

As suggested by Oliveira et al. [15], methanolic extracts have greater antioxidant activity compared to ethanolic and water based. The antioxidant activity values of methanolic extracts of *A. unedo* were determined during a period of 12 months (Figure 1).

The results of the antioxidant activity vary with the month observed and cannot be attributed to any of the analyzed polyphenols individually (*r* ^2^ < 0.25); rather it presents the overall activity of the total extracted polyphenols including phenolic acids and flavonoids other than analyzed chlorogenic acid, quercitrin, isoquercitrin and hyperoside. These flavonoids are attributed up to only 12% of total flavonoids.

Specific pharmacological activity is usually the result of a specific substance; for example, the inhibition of 3-hydroxy-3-methylglutaryl coenzyme A reductase can be attributed to quercetin, hyperoside, rutin, and chlorogenic acid fractions of *Crataegus pinnatifida *Bge. [16]. Thus, to achieve a specific action of the extracts of *A. unedo,* leaves should be collected during the months when the concentration of individual substance responsible for specific action is the highest. This means, based on the results obtained, that the leaves should be collected in January to obtain the highest concentrations of hyperoside and quercitrin (0.35 mg/g and 1.94 mg/g, resp.), in June, July, and October for chlorogenic acid (1.45–1.46 mg/g), and for the fraction of quercitrin and isoquercitrin in November (1.98 mg/g and 0.33 mg/g, resp.).

## 4. Conclusion

Different studies have shown beneficial effects of *A. unedo* for human health and suggested the usage of standardized extracts in medicinal products. Although antioxidant activity well correlates with the total content of polyphenols, phenolic acids, and flavonoids [15], a specific action, for example, an antiaggregatory [6] or an anti-inflammatory action [7], is probably the consequence of specific compound(s) to which extracts should be standardized. For this purposes the thin layer chromatography presents simple and readily available technique.

## Figures and Tables

**Table 1:** Parameters of identification of the polyphenols analyzed.

Polyphenol	*R*_*F*_	Color under *λ* = 366 nm
Quercitrin	0.80	Yellow-green
Isoquercitrin	0.64	Yellow-green
Hyperoside	0.57	Yellow-green
Chlorogenic acid	0.49	Blue

**Table 2:** Content of individual polyphenol during the year in the leaves of *A. unedo* expressed in mg per g of dry sample.

	Quercitrin	Isoquercitrin	Hyperoside	Chlorogenic acid
January	1.94 ± 0.08	nd	0.35 ± 0.03	0.70 ± 0.09
February	1.34 ± 0.22	nd	0.12 ± 0.03	0.76 ± 0.09
March	1.38 ± 0.21	nd	nd	1.06 ± 0.19
April	1.38 ± 0.17	nd	0.11 ± 0.03	1.13 ± 0.07
May	1.21 ± 0.05	nd	0.11 ± 0.02	1.06 ± 0.12
June	2.20 ± 0.17	0.07 ± 0.01	0.11 ± 0.04	1.45 ± 0.34
July	1.56 ± 0.09	nd	nd	1.46 ± 0.13
August	1.78 ± 0.30	0.09 ± 0.05	0.21 ± 0.01	1.11 ± 0.14
September	1.74 ± 0.14	nd	nd	0.79 ± 0.16
October	1.78 ± 0.04	nd	nd	1.45 ± 0.59
November	1.98 ± 0.21	0.33 ± 0.08	0.16 ± 0.01	0.76 ± 0.11
December	1.46 ± 0.26	0.13 ± 0.09	0.24 ± 0.01	0.61 ± 0.05

Results expressed as mean ± standard deviation.

nd: not detected.
